# A Unique and Simple Approach to Improve Sensitivity in ^15^N-NMR Relaxation Measurements for NH_3_^+^ Groups: Application to a Protein-DNA Complex

**DOI:** 10.3390/molecules22081355

**Published:** 2017-08-15

**Authors:** Dan Nguyen, Ganesh L. R. Lokesh, David E. Volk, Junji Iwahara

**Affiliations:** 1 Department of Biochemistry and Molecular Biology, Sealy Center for Structural Biology and Molecular Biophysics, University of Texas Medical Branch, Galveston, TX 77555, USA; danguye1@utmb.edu; 2 McGovern Medical School, Brown Foundation Institute of Molecular Medicine for the Prevention of Human Diseases, University of Texas Health Science Center, Houston, TX 77030, USA; lokesh.rao@uth.tmc.edu (G.L.R.L.); david.volk@uth.tmc.edu (D.E.V.)

**Keywords:** dynamics, ion pairs, NH_3_^+^ groups, NMR relaxation, protein side chains

## Abstract

NMR spectroscopy is a powerful tool for research on protein dynamics. In the past decade, there has been significant progress in the development of NMR methods for studying charged side chains. In particular, NMR methods for lysine side-chain NH_3_^+^ groups have been proven to be powerful for investigating the dynamics of hydrogen bonds or ion pairs that play important roles in biological processes. However, relatively low sensitivity has been a major practical issue in NMR experiments on NH_3_^+^ groups. In this paper, we present a unique and simple approach to improve sensitivity in ^15^N relaxation measurements for NH_3_^+^ groups. In this approach, the efficiency of coherence transfers for the desired components are maximized, whereas undesired anti-phase or multi-spin order components are purged through pulse schemes and rapid relaxation. For lysine side-chain NH_3_^+^ groups of a protein-DNA complex, we compared the data obtained with the previous and new pulse sequences under the same conditions and confirmed that the ^15^N relaxation parameters were consistent for these datasets. While retaining accuracy in measuring ^15^N relaxation, our new pulse sequences for NH_3_^+^ groups allowed an 82% increase in detection sensitivity of ^15^N longitudinal and transverse relaxation measurements.

## 1. Introduction

NMR spectroscopy is one of the most powerful techniques for studying protein dynamics. NMR studies have revealed the functional importance of structural dynamics in many biological molecular processes of proteins (e.g., reviewed in Refs [1,2,3,4,5,6,7,8]). While the vast majority of NMR investigations of protein dynamics have probed motions of either backbone NH or side-chain CH_3_ groups, NMR investigations on polar or charged side chains remain rare. Recently, there has been significant progress in NMR methods for investigating the dynamics of charged side chains of proteins [9,10,11,12,13,14,15,16,17]. In particular, NMR methods for Lys side-chain NH_3_^+^ groups have proven to be extremely useful for investigating the dynamics of hydrogen bonding and/or ion pairing [14,15,16,17,18,19,20,21,22,23,24,25].

Lys side-chain NH_3_^+^ groups of proteins undergo rapid hydrogen exchange with water [26,27,28]. As a result of this rapid hydrogen exchange, signals from NH_3_^+^ groups in ^1^H-^15^N heteronuclear single-quantum coherence (HSQC) and heteronuclear multiple-quantum coherence (HMQC) spectra are severely broadened [26]. Importantly, this broadening occurs not only in the ^1^H dimension but also in the ^15^N dimension, because rapid hydrogen exchange greatly enhances scalar relaxation of ^15^N transverse coherence anti-phase with respect to ^1^H (e.g., 2*N_x_H_z_*, 4*N_y_H_z_H_z_*, and 8*N_x_H_z_H_z_H_z_*).

To avoid this problem, Iwahara et al. developed NH_3_^+^-selective heteronuclear in-phase single-quantum coherence (HISQC) and its derivatives [26]. In the HISQC experiment, the in-phase single quantum term *N_x_* or *N_y_* is created at the beginning of the ^15^N evolution period, and in-phase single-quantum coherence *N_+_* (= *N_x_* + *iN_y_*) is maintained via the ^1^H WALTZ decoupling scheme throughout the evolution period. Evolutions to the anti-phase terms such as 2*N_+_H_z_*, 4*N_+_H_z_H_z_*, and 8*N_+_H_z_H_z_H_z_* are suppressed to remove the impact of scalar relaxation on line shape of ^15^N resonances. Scalar relaxation arises from auto-relaxation of the coupled ^1^H nuclei [29,30], and substantially increases the relaxation rates of the 2*N_+_H_z_*, 4*N_+_H_z_H_z_*, and 8*N_+_H_z_H_z_H_z_* terms, compared to the relaxation rates of *N_+_*. The scalar relaxation rate *R_sc_* for each ^1^H nucleus is given by [26]:

(1) $$R_{sc}=\rho_{HH}+k_{ex}^{water}$$

where *ρ_HH_* is the rate for dipole-dipole relaxation with external ^1^H nuclei and $k_{ex}^{water}$ is the rate for hydrogen exchange with water. Scalar relaxation rates for the *N_+_*, 2*N_+_H_z_*, 4*N_+_H_z_H_z_*, and 8*N_+_H_z_H_z_H_z_* terms are 0, *R_sc_*, 2*R_sc_*, and 3*R_sc_*, respectively [31]. Typically, hydrogen exchange is much faster than *ρ_HH_* rates and intrinsic ^15^N relaxation rates for NH_3_^+^ groups [14,15,16,26]. Therefore, rapid hydrogen exchange governs relaxation of the anti-phase terms through the scalar relaxation mechanism and severely broadens ^15^N line shapes of NH_3_^+^ signals in typical 2D ^1^H-^15^N correlation spectra. By maintaining in-phase single-quantum terms *N_x_* and *N_y_*, and thereby removing the scalar relaxation from the *t*_1_ time domain for the ^15^N dimension, the HISQC experiment drastically improved observation of ^1^H-^15^N cross peaks from NH_3_^+^ groups in sensitivity and resolution [26]. Since then, many NMR pulse sequences for NH_3_^+^ groups have implemented the principle of HISQC, and minimized the adverse impacts of scalar relaxation of anti-phase terms with respect to ^1^H nuclei [14,15,16,17,26,32].

Nevertheless, relatively low sensitivity due to rapid hydrogen exchange has been a major practical problem in NMR experiments for Lys side-chain NH_3_^+^ groups of proteins. While some side-chain NH_3_^+^ groups exhibit relatively slow hydrogen-exchange rates due to hydrogen bonds or ion pairs [26,33], many other NH_3_^+^ groups exhibit very rapid hydrogen-exchange rates that severely broaden ^1^H resonances. Due to this problem, NMR experiments on protein side-chain NH_3_^+^ groups are often conducted at relatively low pH (typically pH 4.5–6.0) and low temperature (typically, 2–25 °C) to observe a larger number of signals with stronger intensity [32,34]. In these NMR experiments, co-axial NMR tubes that separate lock solvent (usually, D_2_O) from a sample solution are typically used to avoid isotopically different species (i.e., NDH_2_^+^, and ND_2_H^+^, and ND_3_^+^) of NH_3_^+^ groups. The use of co-axial tubes further decreases sensitivity due to a smaller sample volume and multilayer glass walls. Thus, sensitivity improvement would be desirable for NMR experiments on NH_3_^+^ groups, especially for quantitative experiments such as ^15^N relaxation measurements.

To address these practical needs, we present a unique and simple approach to improve sensitivity in ^15^N relaxation measurements on protein side-chain NH_3_^+^ groups. Our approach involves only minor modifications of the existing pulse sequences. Nevertheless, the pulse sequences implementing this approach significantly improve the detection sensitivity, while maintaining the accuracy in the ^15^N relaxation measurements on NH_3_^+^ groups.

## 2. Results

**Figure 1:**
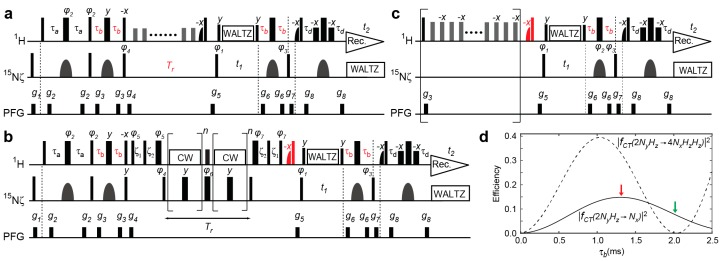
Pulse sequences for the ^15^N relaxation measurement on lysine side-chain NH_3_^+^ groups. The key elements in the current work are indicated in red. Thin and bold bars in black represent hard rectangular 90° and 180° pulses, respectively. Water-selective half-Gaussian (2.1 ms) and soft-rectangular (1.2 ms) 90° pulses are represented by half-bell and short-bold shapes, respectively. Unless indicated otherwise, pulse phases are along *x*, and the carrier position for ^1^H was set to the position of the water resonance. The ^15^N carrier position was set to 33.1 ppm. A gray bell-shape for ^15^N represents an r-SNOB [36] 180° pulse (1.0 ms) selective to Lys side-chain ^15^N_ζ_ nuclei. The delays *τ_a_* and *τ_b_* were 2.7 ms and 1.3 ms, respectively. Quadrature detection in the *t*_1_ domain was achieved using States-TPPI, incrementing the phase *ϕ*_1_. Pulsed field gradients (PFGs) were optimized to minimize the water signal. (**a**) ^15^N *R*_1_ measurement. Although it is not essential owing to negligible CSA-DD cross correlation for NH_3_^+^, a ^1^H 180° pulse, which does not affect H_2_O resonance, was applied every 10 ms during the delay *T_r_* for longitudinal relaxation. Phase cycles: *ϕ*_1_ = (2*y*, 2(−*y*)), *ϕ*_2_ = (*y*, −*y*), *ϕ*_3_ = (4*x*, 4(−*x*)), *ϕ*_4_ = (8*y*, 8(−*y*)), and receiver = (*x*, −*x*, −*x*, *x*, 2(−*x*, *x*, *x*, −*x*), *x*, −*x*, −*x*, *x*); (**b**) ^15^N *R*_2,*ini*_ measurement. The RF strength for ^15^N pulses for the CPMG scheme was 5.4 kHz. The ^1^H carrier position was shifted to 7.8 ppm right after the PFG g_4_ and set back to the position of water resonance right after the PFG g_5_. The RF strength *ω_CW_*/2π of ^1^H CW during the CPMG was set to 4.3 kHz, which was adjusted to satisfy *ω_CW_*/2π = 2*kν_CPMG_* (*k*, integer) [37]. The delays ξ_1_ and ξ_2_ are for alignment of ^1^H magnetization and given by ξ_1_ = 1/*ω_CW_* − (4/π)*τ*_90*H*_ and ξ_2_ = *τ*_90*N*_ − (2/π)*τ*_90*H*_ [37,38], in which *τ*_90_ represents a length of a relevant 90° pulse. Phase cycles: *ϕ*_1_ = (4*y*, 4(−*y*)), *ϕ*_2_ = (8*y*, 8(−*y*)), *ϕ*_3_ = *x*, *ϕ*_4_ = (*x*, −*x*), *ϕ*_5_ = (2*y*, 2(−*y*)), *ϕ*_6_ = (2*x*, 2(−*x*)), *ϕ*_7_ = (2(−y*)*, 2*y*), and receiver = (*x*, −*x*, *x*, −*x*, 2(−*x*, *x*, −*x*, *x*), *x*, −*x*, *x*, −*x*); (**c**) Heteronuclear ^1^H-^15^N NOE measurement. Measurement with ^1^H saturation (5 s) was performed with a train of 180°*x* and 180°(−*x*) pulses (RF strength, 11 kHz) at an interval of 10 ms. The ^1^H carrier position was at 7.8 ppm during the ^1^H saturation period. The reference spectrum was measured without the scheme in the bracket. The recycle delay (including the saturation period) was set to 18 s for a 750-MHz spectrometer. Phase cycles: *ϕ*_1_ = (*y*, −*y*), *ϕ*_2_ = (4*x*, 4*y*, 4(−*x*), 4(−*y*)), *ϕ*_3_ = (2*x*, 2(−*x*)), and receiver = (*x*, −*x*, −*x*, *x*, −*x*, *x*, *x*, −*x*); (**d**) Efficiency in coherence transfers as a function of the delay *τ_b_* calculated using Equations (2) and (3) with |*^1^J_NH_*| = 74 Hz and ^1^H 180° pulse length of 20 μs. The results for the *N_y_* and 4*N_y_H_z_H_z_* terms are shown in solid and dotted lines, respectively. Red and green arrows indicate the values of the delay *τ_b_* in the current and previous pulse sequences, respectively.

Figure 1 shows the optimized NMR pulse sequences for measuring ^15^N *R*_1_ and *R*_2_ relaxation and heteronuclear NOE of NH_3_^+^ groups. In the description below, using the product operator formalism [35] for AX_3_ spin systems, we first explain the previous approach that resolves problems arising from undesired anti-phase or multi-spin-order components of ^15^N magnetizations of NH_3_^+^ groups in ^15^N relaxation measurements. Then, we describe our new approach to improving sensitivity and eliminating undesired components in ^15^N relaxation measurements, showing data that demonstrate the effectiveness of this approach.

### 2.1. Previous and Current Approaches to Eliminating the Adverse Effects of Multi-Spin Order Terms

The first step for measuring ^15^N longitudinal (*R*_1_) and transverse (*R*_2_) relaxation rates is to create the ^15^N in-phase single-quantum term via coherence transfer from ^1^H to ^15^N nuclei through a refocused INEPT scheme [39]. With regard to NH_3_^+^ groups, the product operator terms *N_x_*, 2*N_y_H_z_*, 4*N_x_H_z_H_z_*, and 8*N_y_H_z_H_z_H_z_* are generated in the period of 2*τ_b_* in the first refocused INEPT scheme of our pulse sequence for ^15^N *R*_1_ and *R*_2_ measurements (Figure 1a,b). Because the only term of interest among them is *N_x_*, any effects of the other three terms should be eliminated in these relaxation measurements. The 2*N_y_H_z_* and 8*N_y_H_z_H_z_H_z_* terms are eliminated by the pulsed field gradient (PFG) *g*_4_ after the ^1^H 90°(−*x*) and ^15^N 90°(*y*) pulses at the end of the refocused INEPT scheme. These 90° pulses convert the *N_x_* and 4*N_x_H_z_H_z_* terms into *N_z_* and 4*N_z_H_y_H_y_*, both of which survive the PFG *g*_4_. The 4*N_z_H_y_H_y_* term survives because a PFG alone cannot destroy homonuclear zero-quantum coherence [40]. To avoid any adverse impact of the 4*N_x_H_z_H_z_* term generated in the refocused INEPT scheme, the previous pulse sequences used a value of the time *τ_b_* that erases the 4*N_x_H_z_H_z_* term, but retains the *N_x_* term. This is possible because coherence transfer to these terms depends differently on the time *τ_b_*. The coefficients of these transfers are given by [39]:

(2) $$f_{CT}\left(2N_{y}H_{z}\to N_{x}\right)=\cos^{2}\theta \sin\theta$$

(3) $$f_{CT}\left(2N_{y}H_{z}\to 4N_{x}H_{z}H_{z}\right)=\left(3\cos^{2}\theta -1\right)\sin\theta$$

where $\theta =2\pi J_{NH}\tau_{b}$ and ^1^*J_NH_* represents the one-bond ^1^H-^15^N scalar coupling constant. The use of the time *τ_b_* satisfying $3\cos^{2}\theta -1=0$ thus eliminates the 4*N_x_H_z_H_z_* term, but retains the *N_x_* term [15]. This approach was used for ^13^C *R*_1_ and *R*_2_ relaxation measurements for protein CH_3_ groups as well [41,42]. Because *^1^J_NH_* is typically ~74 Hz for lysine side-chain NH_3_^+^ groups [26], the condition to suppress the 4*N_x_H_z_H_z_* term was achieved using *τ_b_* = 2.1 ms in the original pulse sequences [15]. This condition was also used in the second refocused INEPT scheme for backward coherence transfer, so that any coherence transfer from 4*N_x_H_z_H_z_* to 2*N_y_H_z_* does not contribute to the observed signals. A practical problem in using the condition of $f_{CT}\left(2N_{y}H_{z}\to 4N_{x}H_{z}H_{z}\right)=0$ is that it also reduces $f_{CT}\left(2N_{y}H_{z}\to N_{x}\right)$ from its maximum level, and thereby weakens signals in the ^15^N relaxation measurements for NH_3_^+^ groups (Figure 1d).

In the current work, we eliminate the adverse effects of the 4*N_x_H_z_H_z_* term in a different manner, and maximize $f_{CT}\left(2N_{y}H_{z}\to N_{x}\right)$ to increase sensitivity in ^15^N relaxation measurements for NH_3_^+^ groups. As shown in Figure 1d, the signal arising from the *N_x_* term should be strongest when *τ_b_* = 1.3 ms. Although this condition increases the 4*N_x_H_z_H_z_* term generated through the refocused INEPT scheme, our pulse sequences shown in Figure 1 prevent the undesired 4*N_x_H_z_H_z_* term from becoming observable in the ^1^H detection period *t*_1_. This allows us to use *τ_b_* = 1.3 ms and improve sensitivity without compromising accuracy in ^15^N relaxation measurements.

### 2.2. Assessment of the Sensitivity-Improved ^15^N R_1_ Experiment for NH_3_^+^ Groups

Our pulse sequence for the ^15^N *R*_1_ relaxation measurements on NH_3_^+^ groups is shown in Figure 1a. This pulse sequence is the same as that in Esadze et al. [15], except that the time *τ_b_* is set to 1.3 ms instead of 2.1 ms. The ^1^H 90°(−*x*) and ^15^N 90°(*y*) at the end of the first refocused INEPT convert the *N_x_* and 4*N_x_H_z_H_z_* terms into the *N_z_* and 4*N_z_H_y_H_y_* terms. As mentioned above, both of these terms survive the PFG *g*_4_, and are subjected to the period *T_r_* for relaxation measurement. For measuring ^15^N *R*_1_ relaxation rates, however, only the *N_z_* term should be retained, and any contribution of the 4*N_z_H_y_H_y_* term should be removed. During the period *T_r_*, not only longitudinal relaxation, but also cross-correlation of three ^1^H-^15^N dipole-dipole (DD) relaxation mechanisms occur for the *N_z_* term. The DD-DD cross-correlation causes partial transitions from *N_z_* to 4*N_z_H_z_H_z_* [43]. The composite of water-selective ^1^H 90°(−*x*) and hard ^1^H 90°(*x*) pulses was originally introduced to prevent this term from becoming detectable while maintaining water ^1^H magnetization along +*z* [15]. However, if a considerable amount of the 4*N_z_H_y_H_y_* term is present at the beginning of the period *T_r_*, the composite pulses at the end can partially convert this term into 4*N_z_H_z_H_z_*, which can survive the rest of the pulse sequence and become observable through the second refocused INEPT with *τ_b_* = 1.3 ms. This problem in the ^15^N *R*_1_ measurement can readily be resolved by taking advantage of rapid relaxation of the 4*N_z_H_y_H_y_* term. Due to rapid hydrogen exchange with water, scalar relaxation of anti-phase and multi-spin order terms of ^15^NH_3_^+^ are far faster than the intrinsic ^15^N *R*_1_ and *R*_2_ relaxation of NH_3_^+^ groups [15,26]. Even under the conditions of pH 5.0 and 2 °C, where hydrogen exchange is relatively slow, the relaxation rates of the 4*N_z_H_z_H_z_* term were ~20–100-fold faster than the relaxation rates of the *N_z_* term for the Lys side-chain NH_3_^+^ groups of ubiquitin [15]. The relaxation of the 4*N_z_H_y_H_y_* term should be even faster because of its transverse nature. Therefore, if the minimum duration of period *T_r_* in the ^15^N *R*_1_ relaxation experiment is sufficiently long to let the 4*N_z_H_y_H_y_* term completely decay, the relaxation rates of the *N_z_* term (i.e., ^15^N *R*_1_) can be measured without any adverse contribution from the 4*N_z_H_y_H_y_* term.

**Figure 2:**
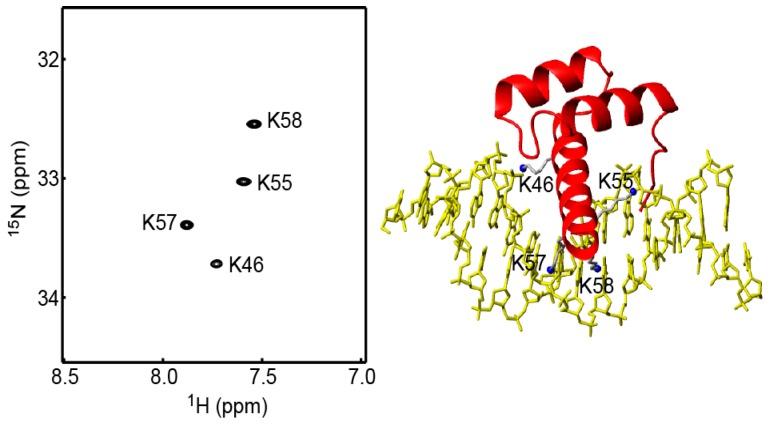
The ^1^H-^15^N HISQC spectrum recorded at 15 °C for the NH_3_^+^ groups in the complex of ^15^N-labeled Antp homeodomain and unlabeled 15-bp DNA containing a phosphorodithioate at the K46 interaction site. The resonance assignment is based on that for the unmodified DNA complex and unique chemical shift perturbation upon site-specific dithioation (i.e., sulfur substitutions of two non-bridging oxygen atoms) of the DNA phosphate at the K46 interaction site [44].

**Figure 3:**
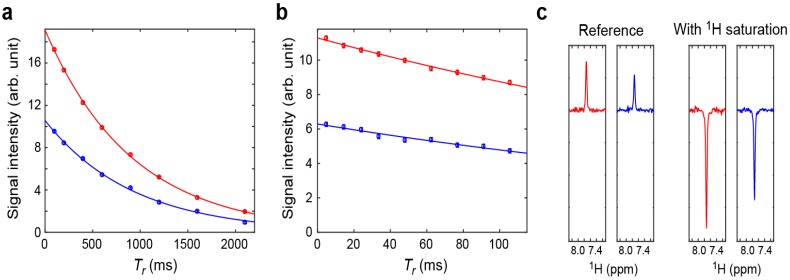
Comparison of the previous [15] and current pulse sequences for measuring ^15^N relaxation of NH_3_^+^ groups. (**a**,**b**) ^15^N longitudinal (Panel **a**) and transverse (Panel **b**) relaxation of the K46 NH_3_^+^ group. The vertical axis represents the signal intensity in the two-dimensional spectra measured as a function of the relaxation period *T_r_*. Solid lines represent the best-fit curves obtained through nonlinear least-squares fitting with a mono-exponential function; (**c**) Slices of the K46 NH_3_^+^ signals along the ^1^H dimension from the two-dimensional spectra with and without ^1^H saturation for the heteronuclear NOE measurements. In each panel, data obtained with the previous and current pulse sequences are shown in blue and red, respectively.

We applied this approach to the Lys side-chain NH_3_^+^ groups of the Antp homeodomain-DNA complex at pH 5.8 and 15 °C. The interfacial Lys side chains K46, K55, K57, and K58 of this protein-DNA complex exhibit well-resolved ^1^H-^15^N cross peaks in the NH_3_^+^-selective ^1^H-^15^N HISQC spectra (Figure 2). For these NH_3_^+^ groups, we measured ^15^N *R*_1_ relaxation rates with the previous and current pulse sequences using the same number of scans and data points. In these ^15^N *R*_1_ measurements, we recorded 2D ^1^H-^15^N spectra using *T_r_* = 100, 200, 400, 600, 900, 1200, 1600, and 2100 ms in an interleaved manner. The minimum duration, *T_r_* = 100 ms, is expected to be long enough to let the 4*N_z_H_y_H_y_* term completely decay through its rapid relaxation. As predicted in Figure 1d, the signals from NH_3_^+^ groups in the spectra recorded with *τ_b_* = 1.3 ms showed significantly stronger intensities than in those recorded with *τ_b_* = 2.1 ms. Figure 3a shows the signal intensity of the K46 NH_3_^+^ group as a function of *T_r_*. The sensitivity was found to improve by a factor of 1.82 on average, which was consistent with the ratio of $|f_{CT}\left(2N_{y}H_{z}\to N_{x}\right){|}^{2}$ at *τ_b_* = 1.3 ms and 2.1 ms. The ^15^N relaxation rates *R*_1_ were determined through nonlinear least-squares fitting with a single exponential function. Table 1 shows the ^15^N *R*_1_ relaxation rates measured with the previous and current pulse sequences for the Lys NH_3_^+^ groups in the Antp homeodomain-DNA complex. The ^15^N *R*_1_ rates from the two experiments were virtually the same, within experimental uncertainties. Not surprisingly, improvement in sensitivity led to higher precision in measured ^15^N *R*_1_ relaxation rates.

### 2.3. Assessment of the Sensitivity-Improved ^15^N R_2_ Experiment for NH_3_^+^ Groups

Our new pulse sequence for ^15^N *R*_2_ measurements is shown in Figure 1b. This pulse sequence differs from our previous one in two ways. First, *τ_b_* = 1.3 ms is used instead of *τ_b_* = 2.1 ms. Second, a composite of water-selective ^1^H 90°(−*x*) and hard ^1^H 90°(*x*) pulses is implemented before the PFG *g*_5_. This additional component is important for canceling the effects of the 4*N_y_H_z_H_z_* term generated through the refocused INEPT scheme. The pulse sequence uses the CW-CPMG scheme together with H_2_O alignment pulse trains [37]. During the ^15^N CPMG spin-echo periods for ^15^N transverse relaxation measurements, a ^1^H continuous wave (CW) is applied at the ^1^H resonances of NH_3_^+^ groups to maintain the in-phase single-quantum term *N_x_* and prevent the anti-phase terms from being produced. Through the ^1^H pulse scheme developed by Hansen et al. [37], water ^1^H magnetization is aligned to the axis of ^1^H CW in the rotating frame to avoid saturation through ^1^H RF inhomogeneity and then is brought back to +*z*. Because this scheme does not align the 4*N_z_H_y_H_y_* term, the terms arising from it are largely purged due to the RF inhomogeneity of the ^1^H CW. However, their component parallel to the CW axis can remain and become the 4*N_z_H_z_H_z_* term through the back-alignment scheme after the period *T_r_*. Unlike the period *T_r_* in the ^15^N *R*_1_ relaxation measurement, the period *T_r_* in the ^15^N *R*_2_ relaxation measurement should be relatively brief, because only a limited number of hard ^15^N 180° pulses can practically be used during the ^15^N CPMG scheme. Therefore, this remaining undesired term cannot be purged completely through relaxation. However, the composite of the water-selective ^1^H 90°(−*x*) and hard ^1^H 90°(*x*) pulses purges this 4*N_z_H_z_H_z_* in the same manner as the 4*N_z_H_z_H_z_* term arising from DD-DD cross-correlation during the period *T_r_* is canceled in the ^15^N *R*_1_ measurement.

For the Lys side-chain NH_3_^+^ groups of the Antp homeodomain in complex with 15-bp DNA, we compared the ^15^N *R*_2_ relaxation data obtained with the old and new pulse sequences under the same conditions. We recorded nine ^1^H-^15^N spectra in an interleaved manner using *T_r_* = 4.8, 14.4, 33.6, 48.0, 76.8, 91.2, and 105.6 ms. Strictly speaking, ^15^N transverse relaxation of NH_3_^+^ groups should occur bi-exponentially due to DD-DD cross-correlation [15,41], but the first 30% decay from the maximum can be treated as a mono-exponential decay, as demonstrated by Esadze et al. [15]. Using mono-exponential fitting, the initial rate constants (*R*_2,*ini*_) for this ^15^N transverse relaxation were determined from the signal intensity as a function of *T_r_*. The results from the data obtained with the previous and current pulse sequences are shown in Figure 3b and Table 1. The *R*_2,*ini*_ rates from these two datasets are in good agreement. Due to the use of *τ_b_* = 1.3 ms, the signal intensities in the spectra recorded with the current pulse sequence were significantly higher than those in the spectra recorded with the previous pulse sequence. As expected, the gain in intensity in the ^15^N *R*_2_ experiment was the same as that in the ^15^N *R*_1_ experiment (i.e., 82% increase on average). This improvement in sensitivity led to significantly higher precision in measured ^15^N *R*_2,*ini*_ rates.

### 2.4. Assessment of the Sensitivity-Improved Heteronuclear NOE Experiment for NH_3_^+^ Groups

Figure 1c shows the pulse sequence for heteronuclear NOE measurements for NH_3_^+^ groups that implements the abovementioned approach. As described by Esadze et al. [15], steady states of the *N_z_* and 4*N_z_H_z_H_z_* terms are created through saturation of ^1^H nuclear magnetization via a train of 180° pulses for heteronuclear NOE measurements on NH_3_^+^ groups. The 4*N_z_H_z_H_z_* steady state occurs due to DD-DD cross-correlation that drives transitions between the *N_z_* and 4*N_z_H_z_H_z_* terms [15]. In the original pulse sequence, *τ_b_* = 2.1 ms was used to avoid any contribution of the 4*N_z_H_z_H_z_* term to the observed signals. However, in the current pulse sequence (Figure 3c), the composite of the water-selective ^1^H 90°(−*x*) and hard ^1^H 90°(*x*) pulses convert the 4*N_z_H_z_H_z_* term into 4*N_z_H_y_H_y_* immediately before the ^15^N 90° pulse leading to the evolution period *t*_1_. As described for the ^15^N *R*_1_ and *R*_2_ experiment, the rest of the pulse sequence does not allow the 4*N_z_H_y_H_y_* term to become observable in the ^1^H detection period *t*_2_. Therefore, the use of *τ_b_* = 1.3 ms improves sensitivity without compromising the quality of heteronuclear NOE data, though the gain in sensitivity is relatively small because there is only a single refocused INEPT scheme in this pulse sequence.

We compared the heteronuclear NOE data obtained with the previous and current pulse sequences for the Lys NH_3_^+^ groups of the Antp homeodomain-DNA complex. Figure 3c shows ^1^H slices of the 2D ^1^H-^15^N spectra recorded with and without ^1^H saturation in the heteronuclear NOE experiments. The heteronuclear NOE values from the datasets obtained with the previous and current pulse sequences agreed well, as shown in Table 1. As expected, the spectra recorded with the new pulse sequence exhibited an increase in the intensity of each signal compared with those recorded with the previous pulse sequence under the same conditions. The improvement in the sensitivity was by a factor of 1.35 on average for the heteronuclear NOE measurements.

## 3. Discussion

As demonstrated above, our new pulse sequences improve sensitivity in ^15^N relaxation measurements on protein side-chain NH_3_^+^ groups without compromising accuracy in measuring intrinsic ^15^N relaxation parameters. By eliminating contributions from the undesired terms and maintaining the maximum level of coherence transfers of the desired terms, this method increased sensitivity by a factor of 1.82 for the *R*_1_ and *R*_2_ experiments and by a factor of 1.35 for the heteronuclear NOE experiment. Although our current paper shows data for a protein-DNA complex only, a similar degree of improvement is expected for other systems of different sizes because Equations (2) and (3) are independent of the molecular rotational correlation time. The sensitivity gains for the ^15^N *R*_1_ and *R*_2_ experiments are larger because these experiments include two refocused INEPT schemes, whereas the heteronuclear NOE experiment has one. In fact, the relative magnitudes of the sensitivity gains (i.e., 1.82 ≈ 1.35^2^) support this explanation. With the current approach, the time for recording the same quality of data can be significantly reduced compared with the previous ^15^N relaxation experiments for NH_3_^+^ groups. The total measurement times for ^15^N *R*_1_ relaxation, *R*_2_ relaxation, and heteronuclear NOE experiments on a 0.8 mM protein-DNA complex (17 kDa) were 18, 20, and 26 h, respectively. Note that signal to noise ratios are proportional to $\sqrt{N_{s}}$, where $N_{s}$ is the number of accumulated scans per free induction decay (FID). To get the same data quality using the previous pulse sequences by increasing the number of scans, the total measurement times would approximately be tripled for ^15^N *R*_1_ and *R*_2_ measurements and doubled for the heteronuclear NOE measurement. Because rapid hydrogen exchange of NH_3_^+^ groups weakens their ^1^H signals, the improvement in sensitivity in these relaxation experiments is practically helpful. We hope that this approach will facilitate NMR studies of dynamic processes involving hydrogen bonds and ion pairs and help advance our understanding of protein dynamics and its functional roles.

## 4. Materials and Methods

The complex of the ^15^N-labeled Antp homeodomain and unlabeled 15-bp DNA was prepared as described in our previous papers [21,25,44]. The DNA phosphate group at the K46 interaction site was dithioated in the chemical synthesis, as previously described [14,25]. A 370-μL solution of 0.8 mM complex in a buffer of 20 mM sodium phosphate (pH 5.8) and 20 mM NaCl was sealed in a 5-mm outer tube of a co-axial NMR tube system. To avoid the deuterated species of NH_3_^+^ groups (i.e., NDH_2_^+^, ND_2_H^+^, and ND_3_^+^), D_2_O for the NMR lock signal was sealed separately in an inter insert of the co-axial tube. The NMR experiments were performed at 15 °C with an Avance III spectrometer (Bruker BioSpin, Fällanden, Switzerland) operated at the ^1^H frequency of 750 MHz. A TCI cryogenic probe was used for NMR detection. The ^1^H and ^15^N acquisition times were 54 ms and 222 ms, respectively. In each experiment, 16 scans were accumulated per FID, and sub-spectra were recorded in an interleaved manner. The NMR data were processed and analyzed using the NMR-Pipe [45] and NMR-View [46] programs. Other experimental details are given in figure captions. The pulse programs and parameter sets for Bruker NMR spectrometers are available upon request via https://scsb.utmb.edu/labgroups/iwahara/software.

## Figures and Tables

**Table 1:** Comparison of ^15^N relaxation parameters measured with the previous and current pulse sequences ^a^. Shown below are data for the Lys side-chain NH_3_^+^ groups in the complex of ^15^N-labeled Antp homeodomain and unlabeled 15-bp DNA containing a phosphorodithioate at the K46 interaction site.

Parameters	K46	K55	K57	K58
^15^N *R*_1_ (s^−1^) ^b^	1.093 ± 0.013	0.637 ± 0.005	1.035 ± 0.004	0.363 ± 0.002
^15^N *R*_1_ (s^−1^) ^c^	1.081 ± 0.023	0.617 ± 0.008	1.037 ± 0.008	0.364 ± 0.003
^15^N *R*_2,*ini*_ (s^−1^) ^b^	2.55 ± 0.10	1.76 ± 0.07	2.95 ± 0.04	1.20 ± 0.03
^15^N *R*_2,*ini*_ (s^−1^) ^c^	2.74 ± 0.20	2.05 ± 0.12	2.76 ± 0.06	1.14 ± 0.06
Heteronuclear NOE ^b^	−2.44 ± 0.12	−2.83 ± 0.10	−2.54 ± 0.05	−2.71 ± 0.05
Heteronuclear NOE ^c^	−2.53 ± 0.18	−2.75 ± 0.13	−2.60 ± 0.08	−2.65 ± 0.07

^a^ The experiments were conducted at 15 °C and the ^1^H frequency of 750 MHz. Uncertainties were estimated using the Monte Carlo approach based on the noise standard deviation of the spectra. ^b^ Measured with the current pulse sequences shown in Figure 1. ^c^ Measured with the previous pulse sequences [15].

## References

[1] Boehr D.D., Dyson H.J., Wright P.E. (2006). An NMR perspective on enzyme dynamics. Chem. Rev..

[2] Clore G.M., Iwahara J. (2009). Theory, practice, and applications of paramagnetic relaxation enhancement for the characterization of transient low-population states of biological macromolecules and their complexes. Chem. Rev..

[3] Kalodimos C.G. (2011). NMR reveals novel mechanisms of protein activity regulation. Protein Sci..

[4] Kay L.E. (2016). New views of functionally dynamic proteins by solution NMR spectroscopy. J. Mol. Biol..

[5] Loria J.P., Berlow R.B., Watt E.D. (2008). Characterization of enzyme motions by solution NMR relaxation dispersion. Acc. Chem. Res..

[6] Palmer A.G. (2001). NMR probes of molecular dynamics: Overview and comparison with other techniques. Annu. Rev. Biophys. Biomol. Struct..

[7] Villali J., Kern D. (2010). Choreographing an enzyme’s dance. Curr. Opin. Chem. Biol..

[8] Wand A.J. (2013). The dark energy of proteins comes to light: Conformational entropy and its role in protein function revealed by NMR relaxation. Curr. Opin. Struct. Biol..

[9] Stafford K.A., Ferrage F., Cho J.H., Palmer A.G. (2013). Side chain dynamics of carboxyl and carbonyl groups in the catalytic function of Escherichia coli ribonuclease H. J. Am. Chem. Soc..

[10] Paquin R., Ferrage F., Mulder F.A., Akke M., Bodenhausen G. (2008). Multiple-Timescale dynamics of side-chain carboxyl and carbonyl groups in proteins by ^13^C nuclear spin relaxation. J. Am. Chem. Soc..

[11] Hansen A.L., Kay L.E. (2011). Quantifying millisecond time-scale exchange in proteins by CPMG relaxation dispersion NMR spectroscopy of side-chain carbonyl groups. J. Biomol. NMR.

[12] Werbeck N.D., Kirkpatrick J., Hansen D.F. (2013). Probing arginine side-chains and their dynamics with carbon-detected NMR spectroscopy: Application to the 42 kDa human histone deacetylase 8 at high pH. Angew. Chem. Int. Ed. Engl..

[13] Trbovic N., Cho J.H., Abel R., Friesner R.A., Rance M., Palmer A.G. (2009). Protein side-chain dynamics and residual conformational entropy. J. Am. Chem. Soc..

[14] Anderson K.M., Esadze A., Manoharan M., Brüschweiler R., Gorenstein D.G., Iwahara J. (2013). Direct observation of the ion-pair dynamics at a protein-DNA interface by NMR spectroscopy. J. Am. Chem. Soc..

[15] Esadze A., Li D.W., Wang T., Brüschweiler R., Iwahara J. (2011). Dynamics of lysine side-chain amino groups in a protein studied by heteronuclear ^1^H-^15^N NMR spectroscopy. J. Am. Chem. Soc..

[16] Zandarashvili L., Esadze A., Iwahara J. (2013). NMR studies on the dynamics of hydrogen bonds and ion pairs involving lysine side chains of proteins. Adv. Protein Chem. Struct. Biol..

[17] Zandarashvili L., Li D.W., Wang T., Brüschweiler R., Iwahara J. (2011). Signature of mobile hydrogen bonding of lysine side chains from long-range ^15^N-^13^C scalar *J*-couplings and computation. J. Am. Chem. Soc..

[18] Chen C.Y., Esadze A., Zandarashvili L., Nguyen D., Pettitt B.M., Iwahara J. (2015). Dynamic equilibria of short-range electrostatic interactions at molecular interfaces of protein-DNA complexes. J. Phys. Chem. Lett..

[19] Iwahara J., Esadze A., Zandarashvili L. (2015). Physicochemical properties of ion pairs of biological macromolecules. Biomolecules.

[20] Esadze A., Chen C., Zandarashvili L., Roy S., Pettitt B.M., Iwahara J. (2016). Changes in conformational dynamics of basic side chains upon protein-DNA association. Nucleic Acids Res..

[21] Nguyen D., Zandarashvili L., White M.A., Iwahara J. (2016). Stereospecific effects of oxygen-to-sulfur substitution in DNA phosphate on ion pair dynamics and protein-DNA affinity. Chembiochem.

[22] Zandarashvili L., Esadze A., Kemme C.A., Chattopadhyay A., Nguyen D., Iwahara J. (2016). Residence times of molecular complexes in solution from NMR data of intermolecular hydrogen-bond scalar coupling. J. Phys. Chem. Lett..

[23] Zandarashvili L., Esadze A., Vuzman D., Kemme C.A., Levy Y., Iwahara J. (2015). Balancing between affinity and speed in target DNA search by zinc-finger proteins via modulation of dynamic conformational ensemble. Proc. Natl. Acad. Sci. USA.

[24] Zandarashvili L., Iwahara J. (2015). Temperature dependence of internal motions of protein side-chain NH_3_^+^ groups: Insight into energy barriers for transient breakage of hydrogen bonds. Biochemistry.

[25] Zandarashvili L., Nguyen D., Anderson K.M., White M.A., Gorenstein D.G., Iwahara J. (2015). Entropic enhancement of protein-DNA affinity by oxygen-to-sulfur substitution in DNA phosphate. Biophys. J..

[26] Iwahara J., Jung Y.S., Clore G.M. (2007). Heteronuclear NMR spectroscopy for lysine NH_3_ groups in proteins: Unique effect of water exchange on ^15^N transverse relaxation. J. Am. Chem. Soc..

[27] Liepinsh E., Otting G. (1996). Proton exchange rates from amino acid side chains—Implications for image contrast. Magn. Reson. Med..

[28] Segawa T., Kateb F., Duma L., Bodenhausen G., Pelupessy P. (2008). Exchange rate constants of invisible protons in proteins determined by NMR spectroscopy. Chembiochem.

[29] Abragam A. (1961). Thermal relaxation in liquids and gases. The Principle of Nuclear Magnetism.

[30] Bax A., Ikura M., Kay L.E., Torchia D.A., Tschudin R. (1990). Comparison of different modes of two-dimensional reverse-correlation NMR for the study of proteins. J. Magn. Reson..

[31] Ollerenshaw J.E., Tugarinov V., Kay L.E. (2003). Methyl TROSY: Explanation and experimental verification. Magn. Reson. Chem..

[32] Esadze A., Zandarashvili L., Iwahara J. (2014). Effective strategy to assign ^1^H-^15^N heteronuclear correlation NMR signals from lysine side-chain NH_3_^+^ groups of proteins at low temperature. J. Biomol. NMR.

[33] Poon D.K., Schubert M., Au J., Okon M., Withers S.G., McIntosh L.P. (2006). Unambiguous determination of the ionization state of a glycoside hydrolase active site lysine by ^1^H-^15^N heteronuclear correlation spectroscopy. J. Am. Chem. Soc..

[34] Wüthrich K. (1986). NMR of Proteins and Nucleic Acids.

[35] Sørensen O.W., Eich G.W., Levitt M.H., Bodenhausen G., Ernst R.R. (1983). Product operator-formalism for the description of NMR pulse experiments. Prog. Nucl. Magn. Reson. Spectrosc..

[36] Kupče E., Boyd J., Campbell I.D. (1995). Short selective pulses for biochemical applications. J. Magn. Reson. Ser. B.

[37] Hansen D.F., Vallurupalli P., Kay L.E. (2008). An improved ^15^N relaxation dispersion experiment for the measurement of millisecond time-scale dynamics in proteins. J. Phys. Chem. B.

[38] Hansen D.F., Kay L.E. (2007). Improved magnetization alignment schemes for spin-lock relaxation experiments. J. Biomol. NMR.

[39] Ernst R.R., Bodenhausen G., Wokaun A. (1987). Heteronuclear polarization transfer. Principles of Nuclear Magnetic Resonance in One and Two Dimensions.

[40] Van de Ven F.J.M. (1995). Dephasing coherences. Multidimensional NMR in Liquids: Basic Principles and Experimental Methods.

[41] Kay L.E., Bull T.E., Nicholson L.K., Griesinger C., Schwalbe H., Bax A., Torchia D.A. (1992). The measurement of heteronuclear transverse relaxation-times in AX3 spin systems via polarization-transfer techniques. J. Magn. Reson..

[42] Palmer A.G., Wright P.E., Rance M. (1991). Measurement of relaxation-time constants for methyl-groups by proton-detected heteronuclear NMR spectroscopy. Chem. Phys. Lett..

[43] Kumar A., Grace R.C.R., Madhu P.K. (2000). Cross-Correlations in NMR. Prog. NMR Spect..

[44] Anderson K.M., Nguyen D., Esadze A., Zandrashvili L., Gorenstein D.G., Iwahara J. (2015). A chemical approach for site-specific identification of NMR signals from protein side-chain NH_3_^+^ groups forming intermolecular ion pairs in protein-nucleic acid complexes. J. Biomol. NMR.

[45] Delaglio F., Grzesiek S., Vuister G.W., Zhu G., Pfeifer J., Bax A. (1995). NMRPipe: A multidimensional spectral processing system based on UNIX pipes. J. Biomol. NMR.

[46] Johnson B.A., Blevins R.A. (1994). NMR view: A computer-program for the visualization and analysis of NMR data. J. Biomol. NMR.

